# Cross-resistance and synergism bioassays suggest multiple mechanisms of pyrethroid resistance in western corn rootworm populations

**DOI:** 10.1371/journal.pone.0179311

**Published:** 2017-06-19

**Authors:** Adriano E. Pereira, Dariane Souza, Sarah N. Zukoff, Lance J. Meinke, Blair D. Siegfried

**Affiliations:** 1 Division of Plant Sciences, University of Missouri, Columbia, Missouri, United States of America; 2 Department of Entomology, University of Nebraska, Lincoln, Nebraska, United States of America; 3 Southwest Research and Extension Center, Kansas State University, Garden City, Kansas, United States of America; 4 Department of Entomology & Nematology, University of Florida, Gainesville, Florida, United States of America; Universidade Federal de Vicosa, BRAZIL

## Abstract

Recently, resistance to the pyrethroid bifenthrin was detected and confirmed in field populations of western corn rootworm, *Diabrotica virgifera virgifera* LeConte from southwestern areas of Nebraska and Kansas. As a first step to understand potential mechanisms of resistance, the objectives of this study were i) to assess adult mortality at diagnostic concentration-LC_99_ to the pyrethroids bifenthrin and tefluthrin as well as DDT, ii) estimate adult and larval susceptibility to the same compounds as well as the organophosphate methyl-parathion, and iii) perform synergism experiments with piperonyl butoxide (PBO) (P450 inhibitor) and S,S,S-tributyl-phosphorotrithioate (DEF) (esterase inhibitor) in field populations. Most of the adult field populations exhibiting some level of bifenthrin resistance exhibited significantly lower mortality to both pyrethroids and DDT than susceptible control populations at the estimated LC_99_ of susceptible populations. Results of adult dose-mortality bioassays also revealed elevated LC_50_ values for bifenthrin resistant populations compared to the susceptible control population with resistance ratios ranging from 2.5 to 5.5-fold for bifenthrin, 28 to 54.8-fold for tefluthrin, and 16.3 to 33.0 for DDT. These bioassay results collectively suggest some level of cross-resistance between the pyrethroids and DDT. In addition, both PBO and DEF reduced the resistance ratios for resistant populations although there was a higher reduction in susceptibility of adults exposed to PBO versus DEF. Susceptibility in larvae varied among insecticides and did not correlate with adult susceptibility to tefluthrin and DDT, as most resistance ratios were < 5-fold when compared to the susceptible population. These results suggest that both detoxifying enzymes and target site insensitivity might be involved as resistance mechanisms.

## Introduction

The western corn rootworm (WCR), *Diabrotica virgifera virgifera* LeConte, is considered the most important insect pest of field corn (*Zea mays* L.) in the U.S. Corn Belt with estimates of over $1 billion in yield loss and control expenditures annually [1–4]. Injury caused by WCR to corn plants is due to larval feeding on the roots, which reduces water and nutrient uptake, and compromises plant stability [1, 5, 6]. At high infestation levels, injured plants become lodged during strong rain or wind events making the plants difficult to harvest. The WCR has evolved resistance to many control methods including insecticides, crop rotation, and corn hybrids expressing *Bacillis thuringiensis* Berliner (Bt) toxins [7–13] making sustainable management difficult.

In the western U.S. Corn Belt, continuous corn is the predominant cropping pattern and often the most profitable under irrigation; crop rotation options are limited. This practice often leads to build-up of WCR densities and annual pest challenges [14] (Meinke et al. 2009). Therefore, in this system, a combination of tactics is needed to effectively manage rootworms. Bt corn expressing rootworm-active Cry toxins has been widely adopted in this region but resistance evolution to some traits has reduced the effectiveness of this management tactic [12]. Therefore, insecticides are important complementary tactics to Bt corn to prevent silk-clipping or reduce rootworm densities [15] (Meinke 2014).

There is a long history of insecticide use to manage the WCR in Nebraska. The chlorinated hydrocarbon DDT was the first synthetic insecticide to be tested and recommended for WCR adult control [16–18]. It was also commonly used for other pests and applied as a spray, dust, or soil insecticide [16, 19]. The extent and duration of DDT use for WCR control is unclear. However, it is well documented that the cyclodiene insecticide aldrin replaced DDT in WCR management programs during the 1950’s and was widely used through the 1960s in Nebraska [18]. This may have been due to higher WCR susceptibility and reduced amounts of aldrin necessary for control [16, 17, 19, 20]. Because of rapid development of resistance to cyclodiene insecticides, organophosphates and carbamates became widely used for WCR control [21]. The use of both insecticide classes for corn rootworm management has since been restricted because of their common mode of action as acetylcholinesterase inhibitors and potential risks to human health [22–24].Pyrethroid insecticides remain one of the few chemical options to control corn rootworms both as soil insecticides which target larvae and as adulticides to prevent silk-clipping or reduce oviposition [21, 25]. This has resulted in widespread use of pyrethroids, sometimes with multiple applications in a single growing season. In areas of western Nebraska and southwestern Kansas, reports of inadequate rootworm control with the pyrethroid insecticide, bifenthrin, have been increasing in recent years (SNZ and LJM, personal communication). Pereira et al. [10] reported significant differences in adult WCR mortality among U.S. populations when bioassayed at the diagnostic bifenthrin concentration (LC_99_ = 0.77 μg/vial), with western Corn Belt populations exhibiting the lowest susceptibility, especially in southwestern Nebraska and southwestern Kansas. The highest adult LC_50_s were observed in populations from Keith and Chase Counties, NE, and Finney County, KS, which were 5–10 times greater than a susceptible laboratory non-diapause population [10]. Similar relationships between larval progeny from these populations and the standard lab control colony in larval bioassays indicated that susceptibility differences were a heritable trait. These results provided initial evidence that resistance to bifenthrin has evolved in field populations that have been exposed for multiple years to pyrethroid insecticides.

To obtain preliminary information on possible resistance mechanisms we performed a set of experiments to determine the cross-resistance patterns among populations previously shown to exhibit bifenthrin resistance. Pyrethroids are classified based on the absence (type I) or presence (type II) of a α-cyano-group in the phenoxybenzil moiety [26–28]. Pyrethroids and DDT act on the same voltage-gated sodium channel in the insect nervous system, and cross-resistance between these two insecticide classes has been shown to be common among many insect pests. Because of reduced knockdown after initial resistance, this type of resistance is commonly referred to as knockdown resistance (*Kdr*) [29–32]. To determine if enhanced detoxification could also be involved as a resistance mechanism, synergism bioassays were performed with known inhibitors of metabolic detoxification [33]. Resistance in WCR to methyl parathion was reported in Nebraska during the 1990s [34] and a key mechanism involved was enhanced enzyme activity, especially esterases [35]. Because this resistance has been maintained in field populations after removal of selection pressure [36], susceptibility of both larvae and adults to this compound were characterized in this study to complement synergism bioassays to investigate if enhanced enzyme activity may be contributing to the pyrethroid resistance mechanism.

The objectives of this study were to 1) compare WCR adult and larval susceptibility (LC_50_’s) from laboratory and field populations to tefluthrin, bifenthrin, DDT and methyl parathion, 2) compare adult susceptibility to a diagnostic concentration of bifenthrin, tefluthrin, and DDT, and 3) compare WCR adult susceptibility to tefluthrin with and without the synergists piperonyl butoxide (PBO) and S,S,S-tributyl-phosphorotrithioate (DEF). These data will be important to guide future molecular studies towards the elucidation of the biochemical and genetic basis of resistance.

## Materials and methods

### WCR populations

**Fig 1:**
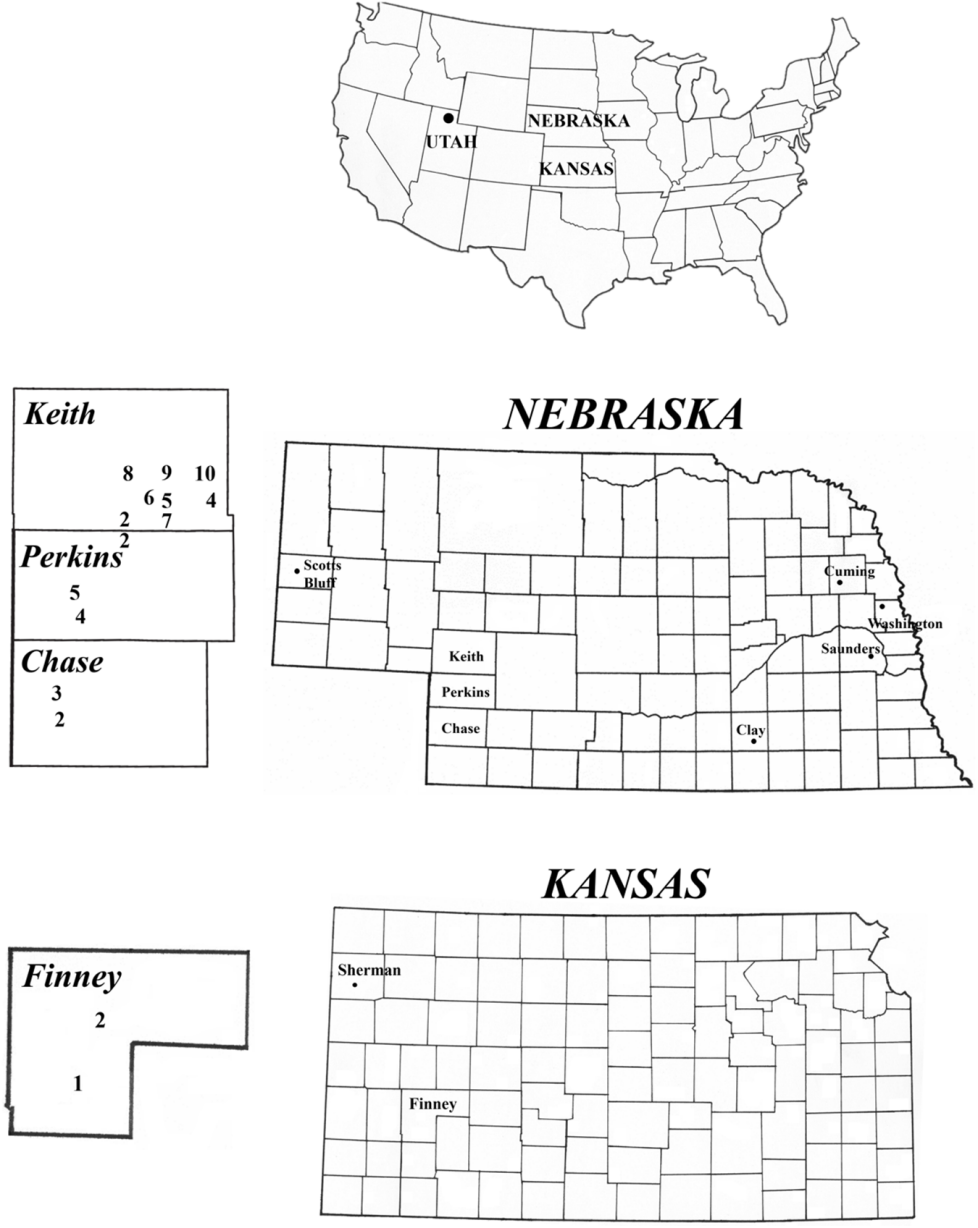
U.S. map showing states Utah, Nebraska (counties Keith, Perkins, Chase, Saunders, Clay, Cuming, and Washington), and Kansas (counties Sherman and Finney) where western corn rootworm beetles were collected for diagnostic concentration and susceptibility bioassays. Numbers in the enlarged counties Keith, Perkins, and Chase in Nebraska, and Finney in Kansas, correspond to population numbers listed from these counties in Fig 2 and Tables 2–4 and show spatially where collections were made. Dots within remaining counties and Utah also show spatial locations of collections.

Adult WCR field populations were collected in Nebraska (2014: Perkins, Chase, Keith, Clay, and Saunders Counties; 2015: Keith, Chase, Perkins, Saunders, Scotts Bluff Counties) and Kansas (2014: Sherman and Finney Counties) (Fig 1), from field corn and blooming weeds using aspirators or sweep nets. WCR collections were returned to the laboratory and maintained in Bugdorm^®^ cages (30 x 30 x 30 cm) with fresh sweet corn ears for at least 24 h before initiating bioassays. Perkins, Keith, and Chase Counties in Nebraska, and Finney County in Kansas were considered problem areas as significantly reduced susceptibility at a diagnostic concentration of bifenthrin was observed in both 2013 and 2014 [10]. Control populations included a non-diapause susceptible population provided by Crop Characteristics^®^ LLC. (Farmington, MN), and field susceptible populations from Saunders and Scotts Bluff counties in Nebraska. Clay County, NE was included in 2014 collections as a population exhibiting intermediate susceptibility to bifenthrin between problem county populations and susceptible populations. Field collections were allowed by the owners (private or University) and collections from Kansas were shipped with APHIS-USDA permits (No. P526P-14-03957 and P526P-15-01279). This research project did not involve any endangered or protected species.

WCR eggs were obtained from adult field population collections (2014: NE: Perkins, Clay, Saunders, Washington, Cuming Counties; UT: Cache County; KS: Finney, Sherman Counties; 2015: NE: Keith County), stored under conditions to facilitate survival/diapause completion [37] and then neonate larvae were bioassayed the following year. Larvae from the Crop Characteristics^®^ LLC non-diapause WCR colony were used as the standard control. Eggs from the non-diapause colony were held at 25^°^C until hatch.

### Adult diagnostic concentration (LC_99_) and baseline susceptibility (LC_50_) bioassays

**Fig 2:**
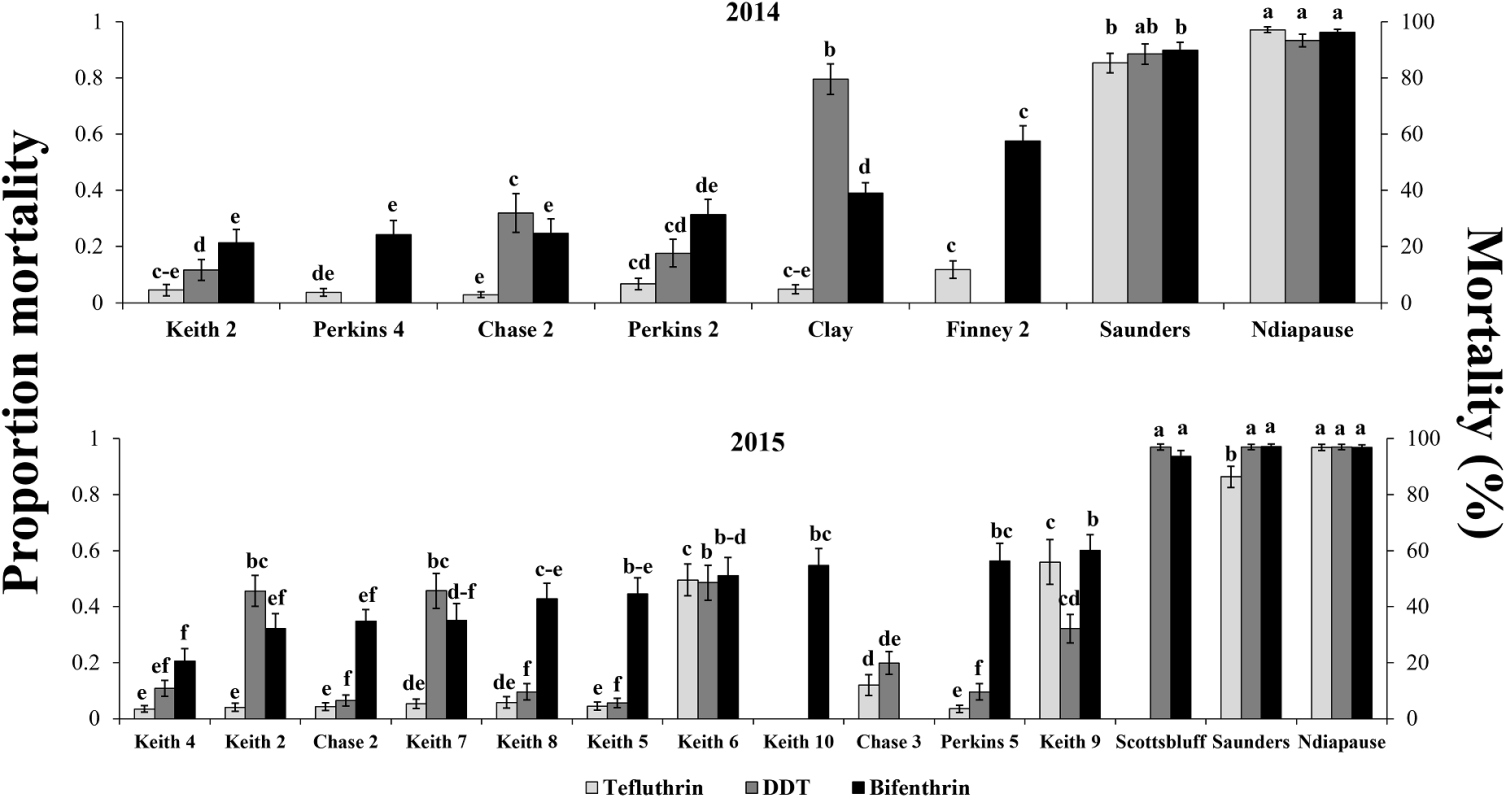
Mean proportion western corn rootworm adult mortality (± SE) at diagnostic concentration (LC_99_) of field populations and a susceptible non-diapause population in 2014 and 2015 bioassays for the insecticides bifenthrin, tefluthrin, and DDT. N = at least 10 reps of 10 beetles per bioassay; LC_99_: Bifenthrin = 0.77 μg/vial; Tefluthrin = 0.60 μg/vial; DDT = 6.02 μg/vial. Within insecticides, means with the same letter above bars are not significantly different (LSmeans test, p>0.05). Bifenthrin data for 2014 taken from Pereira et al. (2015).

All the populations tested at the diagnostic concentration (LC_99_) for the three insecticides were collected in 2014 and 2015 from problem areas in western Nebraska and Kansas identified with an LC_99_ in 2013 and 2014 [10]. Bifenthrin data from 2014 collections is published in Pereira et al [10] and is presented in Fig 2 to facilitate comparison of susceptibility with the other two insecticides. Two susceptible field populations, from Saunders (2014 and 2015) and Scotts Bluff Counties (2015), were also tested. The diagnostic concentration (LC_99_) value for bifenthrin (0.77 μg/vial; Table 1) was generated for WCR adults from 10 laboratory populations known to be susceptible to bifenthrin as described in Pereira et al. [10]. The diagnostic LC_99_ values for the insecticides tefluthrin (0.60 μg/vial) and DDT (6.02 μg/vial) (Table 1) were generated using unsexed non-diapause susceptible beetles < 48 h-old purchased from Crop Characteristics^®^ LLC. (Farmington, MN). Diagnostic concentrations for each insecticide were obtained from bioassays using 20 ml glass scintillation vials containing 10 adults each, and 6–8 concentrations made in 2x series dilutions (bifenthrin- from 0.0625 to 8.0 μg/vial; tefluthrin- from 0.03125 to 4.0 μg/vial; DDT- from 0.125 to 16.0 μg/vial) and three replicates per concentration.

**Table 1:** Baseline susceptibility and diagnostic concentration (LC_99_) (± 95% confidence interval) of western corn rootworm adults by contact to bifenthrin, tefluthrin, and DDT generated from laboratory colonies.

Insecticides	N^a^	Slope (±SE)	LC_99_ (95% CI) μg/vial	*X*^*2*^ (d.f.)
Bifenthrin^b^	2100	2.68 (0.12)	**0.77** (0.53–1.34)	12.4 (4)
Tefluthrin	180	8.26 (1.60)	**0.60** (0.46–0.88)	0.03 (3)
DDT	240	3.30 (0.40)	**6.02** (2.98–53.7)	15.9 (5)

^a^Number of adults tested

^b^Bifenthrin data from Pereira et al. [10]

Diagnostic bioassays were performed in 20 ml glass scintillation vials following the same methodology described in Pereira et al. [10], consisting of at least 10 replicates plus control with each vial containing 10 adults and mortality evaluated after 24 h. The exception was DDT, in which adult mortality in bioassays was recorded after 48 h as mortalities were observed to be slower when compared to other insecticides. Adult populations from eight sites in southeastern Keith County (Fig 1) were bioassayed at LC_99_ to evaluate potential spatial variability in susceptibility to bifenthrin among populations given the low susceptibility of Keith 1–3 to bifenthrin found in 2013 and 2014 [10].

To compare susceptibility based on LC_50_ values and allow calculation of resistance ratios (RR), three field populations from Keith County that exhibited reduced susceptibility at the diagnostic concentrations (labeled 2, 4, and 5), one laboratory susceptible and one field population considered to be susceptible (Saunders County) were bioassayed in 2015 using residual vial bioassays with bifenthrin, tefluthrin, DDT, and methyl parathion. Bioassay methodology was the same as previously described to obtain LC_99_ values. Concentrations used for methyl parathion were 2x series dilutions ranging from 0.03125 to 2.0 μg/vial.

### Adult synergism bioassays

Adult synergism bioassays were conducted with tefluthrin because RRs were larger than bifenthrin in dose-mortality bioassays which provided a greater opportunity to evaluate potential effects of synergists. PBO and DEF were used as cytochrome P-450-dependent monooxygenase and hydrolase inhibitors, respectively, in synergism bioassays. The technical grade synergists (> 98%) were purchased from Chem Service, Inc. (West Chester, PA). Synergists were applied topically in 0.5 μl acetone containing either 2 μg of PBO or 0.2 μg of DEF to the ventral thorax of WCR beetles using a micro-syringe (Hamilton^®^, Reno, NV); control beetles received only 0.5 μl of acetone. Beetles were treated 1.5 h prior to transfer to insecticide-treated vials [33] and mortality was recorded after 24 h. WCR beetles that did not respond within 20 seconds to probing or were unable to right themselves when placed ventral side up, were considered dead.

### Larval bioassays

Larval susceptibility was characterized for bifenthrin, tefluthrin, DDT, and methyl parathion during 2015 for the susceptible non-diapause population and most of the 11 field populations collected as adults in 2014 except where egg availability limited the number of bioassays that could be conducted (i.e., Perkins 1, Finney 2, and Sherman). Larval bioassays with progeny of field populations Keith 2, 4, and 5 collected in 2015, were also performed with all four insecticides in 2016, including the susceptible non-diapause colony to enable comparison of LC_50_s from adult and larval bioassays within populations. In each bioassay, neonate larvae < 36 h-old were exposed to 5–8 increasing insecticide concentrations made in 2x series dilution (bifenthrin- from 0.0625 to 8.0 ng/cm^2^; tefluthrin- from 0.125 to 8.0 ng/cm^2^; DDT- from 0.015625 to 4.0 ng/cm^2^; methyl parathion- from 0.03125 to 0.5 ng/cm^2^) applied to filter paper placed in 5 cm petri dishes for each insecticide and mortality was recorded after 24 h following methods described in Pereira et al. [10] and Magalhaes et al. [38]. LC_50_ values were generated for the susceptible non-diapause population in 2015 and 2016 and RRs were calculated by dividing the LC_50_ value for each field population by the non-diapause population LC_50_ for each respective year. Technical grade > 98% bifenthrin, methyl parathion, and DDT were purchased from Chem Service, Inc. (West Chester, PA), and ≥ 95% tefluthrin was provided by Santa Cruz Biotechnology, Inc. (Dallas, TX).

### Statistical analysis

Data from adult and larval concentration-mortality curve bioassays was used in statistical analyses if mortality in the susceptible control populations was < 20%. The LC_50_ values were obtained by probit analysis [39] and calculated using the POLOPlus-PC software [40]. RRs were calculated by dividing the LC_50_ of each field population by the LC_50_ of the non-diapause susceptible population by using POLOPlus-PC software. Confidence intervals for RRs were generated by POLOPlus-PC software as described by Robertson et al. [41] and compared to test the significance of differences among populations at the 95% level of confidence. With this test, if the 95% confidence interval calculated for a ratio does not include 1.0, a significant difference exists between the values being compared [41, 42]. The statistical methods described above were also used to calculate LC_50_s and RRs for synergism bioassay data.

Proportion survival data from diagnostic bioassays were fit to a Beta-binomial distribution [43,44] with a logit link function and then a generalized linear mixed model using PROC GLIMMIX in SAS was performed (software 9.3, SAS Institute, Cary, NC) to compare survival among populations. Separate analyses were conducted for each insecticide tested. Mean separation was determined by LSMEANS comparisons at α<0.05. Non-transformed means (±SE) are reported in the paper.

## Results

### WCR adult mortality at diagnostic concentrations (LC_99_)

During 2014 and 2015, adult mortalities of all field populations to bifenthrin, tefluthrin, and DDT bioassays were statistically lower than the susceptible non-diapause and susceptible field populations collected from Saunders County, NE except for the Clay County population in the 2014 DDT bioassay (Fig 2). In general, reduced susceptibility among the two pyrethroids and DDT was often observed within populations which suggests cross-resistance among the three compounds in both years (Fig 2). Within insecticides, significant variation in mortality at LC_99_ occurred among some populations but evidence of bifenthrin and tefluthrin resistance and a reasonable level of cross-resistance with DDT was consistent across populations bioassayed from three adjoining Nebraska counties (Figs 1 and 2).

### Adult baseline susceptibility bioassay

Results indicate reduced susceptibility among the pyrethroids and DDT in the three Keith populations bioassayed when compared to the susceptible non-diapause population with RRs ranging from 2.5 to 5.5-fold for bifenthrin, 28 to 54.8-fold for tefluthrin, and 16.3 to 33.0 for DDT (Table 2). These results support the generally lower mortality of these populations when tested at LC_99_ for each population (Fig 2). For bifenthrin, RRs were <10-fold and similar to results presented by Pereira et al. [10]. RRs for the susceptible field population from Saunders County were relatively low for all four insecticides ranging from 0.17–2.5 (Table 2). Two of the three Keith populations exhibited elevated RR values in methyl parathion bioassays, with the highest RR 6.43-fold (Table 2).

**Table 2:** WCR adult susceptibility and RRs to four insecticides between field populations collected in 2015 considered resistant (Keith County populations, NE) and susceptible (Saunders Co-NE), including a susceptible non-diapause population.

Population/Insecticides	N^a^	Slope (±SE)	LC_50_ (95% CI) μg/vial	RR_50_^b^ (95% CI)	*X*^2^ (d.f.)
**Non-diapause (lab susceptible)**					
Bifenthrin	210	3.46 (0.48)	0.17 (0.14–0.20)	-	0.87 (4)
Tefluthrin	210	6.03 (1.01)	0.10 (0.09–0.11)	-	0.44 (4)
DDT	240	2.81 (0.32)	0.36 (0.22–0.63)	-	14.6 (5)
Methyl parathion	240	6.47 (1.40)	0.15 (0.12–0.17)	-	0.03 (5)
**Saunders (field susceptible)**					
Bifenthrin	270	2.12 (0.27)	0.05 (0.03–0.07)	0.17 (0.14–0.20)	7.07 (6)
Tefluthrin	210	2.82 (0.34)	0.24 (0.12–0.51)	2.49 (1.94–3.19)	14.9 (4)
DDT	210	4.37 (0.88)	0.49 (0.37–0.59)	1.32 (0.99–1.76)	0.73 (4)
Methyl parathion	240	4.06 (0.62)	0.07 (0.06–0.09)	0.48 (0.37–0.62)	2.70 (5)
**Keith 2 (field resistant)**					
Bifenthrin	240	3.21 (0.39)	0.86 (0.69–1.00)	5.03 (3.86–6.54)	2.54 (5)
Tefluthrin	240	10.52 (2.04)	5.38 (3.96–7.01)	54.8 (45.2–66.4)	8.07 (5)
DDT	210	2.59 (0.34)	5.97 (4.80–7.61)	16.3 (11.6–22.9)	1.25 (4)
Methyl parathion	210	6.07 (1.88)	0.53 (0.36–0.63)	3.49 (2.67–5.24)	3.95 (4)
**Keith 4 (field resistant)**					
Bifenthrin	180	1.41 (0.27)	0.93 (0.64–1.35)	5.63 (3.77–8.42)	1.29 (3)
Tefluthrin	180	4.42 (0.63)	2.76 (2.33–3.25)	28.0 (22.6–34.7)	0.86 (3)
DDT	210	1.41 (0.45)	12.1 (5.17–19.5)	33.1 (19.1–57.3)	0.95 (4)
Methyl Parathion	180	2.83 (0.47)	0.63 (0.51–0.85)	6.43 (4.86–8.52)	0.57 (3)
**Keith 5 (field resistant)**					
Bifenthrin	210	2.72 (0.41)	0.43 (0.32–0.50)	2.60 (1.89–3.59)	2.61 (4)
Tefluthrin	180	4.22 (0.78)	2.89 (2.42–3.59)	29.4 (23.3–37.0)	0.14 (3)
DDT	240	1.53 (0.53)	6.57 (1.53–11.2)	18.0 (9.06–33.9)	3.87 (5)
Methyl Parathion	180	2.39 (0.38)	0.15 (0.07–0.25)	1.53 (1.12–2.08)	4.02 (3)

^a^Number of insects tested

^b^RRs between Keith and non-diapause susceptible populations

### Synergism bioassays

RRs for all treatments between field and susceptible populations were significantly different based on confidence intervals that do not include 1 [40, 41] (Table 3). LC_50_ values calculated for the three resistant populations were much lower after beetles were exposed to PBO when compared to the susceptible non-diapause population, with RRs dropping from 25.6 to 10.3-fold for Keith 2, from 27.8 to 5-fold for Keith 4, and from 14.5 to 3.7-fold for Keith 5 (Table 3). There was also a reduction in the LC_50_ values after beetles were exposed to DEF, with RRs dropping from 25.6 to 15.5-fold for Keith 2, from 27.8 to 10.8-fold for Keith 4, and from 14.5 to 8.4-fold for Keith 5 (Table 3). In general, the effect of DEF on RRs was less than observed for PBO.

**Table 3:** WCR adult susceptibility of field populations to tefluthrin with and without synergists PBO (cytochrome P-450 monooxigenases inhibitor) and DEF (esterases inhibitor) and respective RR.

Population/synergist	N^a^	Slope (±SE)	LC_50_ (95% CI) μg/vial	RR^b^ (95% CI)	*X*^2^ (d.f.)
**Non-diapause (lab susceptible)**					
No synergistic	180	2.33 (0.31)	0.14 (0.06–0.33)	-	7.33 (3)
PBO	180	3.42 (0.45)	0.03 (0.02–0.03)	-	2.97 (3)
DEF	270	5.90 (0.91)	0.19 (0.15–0.24)	-	7.16 (6)
**Keith 2 (field resistant)**					
No synergistic	210	3.65 (0.54)	3.59 (2.13–7.65)	26.0 (18.9–35.7)	12.0 (4)
PBO	180	1.68 (0.26)	0.35 (0.15–1.30)	11.7 (8.98–19.5)	5.98 (3)
DEF	300	5.59 (1.59)	2.33 (1.41–2.85)	12.3 (8.97–17.8)	7.34 (7)
**Keith 4 (field resistant)**					
No synergistic	210	8.03 (1.43)	3.89 (2.87–4.64)	27.8 (21.1–37.5)	5.39 (4)
PBO	180	4.07 (0.87)	0.15 (0.11–0.19)	5.00 (4.12–7.71)	0.86 (3)
DEF	210	3.99 (0.49)	1.62 (1.19–2.17)	8.55 (6.84–10.7)	5.59 (4)
**Keith 5 (field resistant)**					
No synergistic	210	5.27 (0.74)	2.03 (1.74–2.35)	14.7 (10.9–19.8)	2.44 (4)
PBO	210	2.03 (0.27)	0.11 (0.06–0.20)	3.67 (3.12–5.87)	7.90 (4)
DEF	180	3.19 (0.43)	1.26 (0.94–1.58)	6.62 (4.94–8.87)	1.83 (3)

^a^Number of insects tested

^b^Resistance ratios between Keith and non-diapause susceptible populations within the respective

treatment

### WCR larval susceptibility to four insecticides

The larval susceptibility results from 2015 generated with neonates from eggs laid by beetles collected in 2014 exhibited RRs < 5-fold for all four insecticides when compared to the susceptible non-diapause population, except for the Clay County population which was 11.1-fold more tolerant to tefluthrin (Table 4). For populations generally considered to be susceptible to bifenthrin, such as Saunders-NE, Washington-NE, and Cache-UT, RR_50_s were < 3-fold (Table 4). For other field populations considered resistant to pyrethroids, RR_50_s were around 2-3-fold for bifenthrin, < 4-fold for tefluthrin, < 2.2-fold for DDT, and around 2-3-fold for methyl parathion (Table 4). Perkins 4 was the only population considered resistant to be bioassayed for bifenthrin in 2015 (Table 4), and the RR was slightly lower (3.6-fold) than the six populations (Perkins 1 and 2, Finney 1 and 2, Sherman, and Clay) bioassayed in 2014, which had RRs between 4.4 and 8.3-fold [10]. Higher larval RRs were observed for tefluthrin in most of the field populations when compared to the other insecticides except Saunders, Washington, and Cache populations which are considered field susceptible (Table 4).

**Table 4:** WCR larval susceptibility and RRs of field and non-diapause laboratory populations to the insecticides bifenthrin, tefluthrin, DDT, and methyl parathion. Diapause eggs were collected from field populations in 2014 and 2015 (Keith populations) and bioassays performed in 2015 and 2016 (Keith populations).

Population/Insecticides	N^a^	Slope (±SE)	LC_50_ (95% CI) ng/cm^2^	RR_50_^b^ (95% CI)	*X*^2^ (d.f.)
			**2015**		
**Non-diapause (lab susceptible)**					
Bifenthrin	420	3.02 (0.36)	0.58 (0.49–0.68)	-	0.26 (4)
Tefluthrin	420	2.90 (0.32)	0.22 (0.12–0.33)	-	10.9 (4)
DDT	480	2.66 (0.38)	7.54 (5.07–9.92)	-	10.0 (6)
Methyl Parathion	420	3.94 (0.39)	0.06 (0.05–0.08)	-	8.4 (4)
Perkins Co 1-NE* (field resistant)					
Tefluthrin	480	2.74 (0.23)	0.80 (0.38–1.96)	3.60 (2.85–4.55)	34.3 (5)
Perkins Co 2-NE* (field resistant)					
Tefluthrin	420	6.04 (0.85)	0.91 (0.81–1.01)	4.12 (3.33–5.09)	3.44 (4)
DDT	420	2.43 (0.40)	15.7 (10.6–22.5)	2.08 (1.58–2.75)	5.05 (4)
Methyl Parathion	360	5.83 (0.70)	0.09 (0.08–0.10)	1.46 (1.24–1.72)	1.87 (3)
Finney Co 1-KS* (field resistant)					
Tefluthrin	360	3.17 (0.30)	0.82 (0.72–0.94)	3.72 (2.96–4.67)	2.70 (3)
DDT	420	1.35 (0.17)	7.66 (3.89–29.7)	1.02 (0.70–1.47)	12.1 (4)
Methyl Parathion	420	3.75 (0.78)	0.10 (0.04–0.13)	1.66 (1.28–2.16)	5.63 (4)
Finney Co 2-KS* (field resistant)					
DDT	480	1.02 (0.18)	6.27 (3.71–14.2)	0.83 (0.43–1.61)	1.76 (5)
Sherman Co-KS* (field resistant)					
DDT	260	3.79 (0.50)	6.17 (4.96–7.41)	0.82 (0.65–1.04)	0.31 (2)
Clay Co-NE* (field ‘moderate’ resistant)					
Tefluthrin	480	7.42 (1.23)	2.44 (2.18–2.70)	11.1 (8.96–13.64)	4.87 (5)
DDT	360	2.61 (0.38)	16.7 (9.91–29.0)	2.21 (1.70–2.89)	5.65 (3)
Methyl Parathion	360	4.72 (0.65)	0.14 (0.10–0.18)	2.28 (1.90–2.73)	4.14 (3)
**Perkins Co 4-NE (field resistant)**					
Bifenthrin	420	2.18 (0.31)	2.11 (1.57–2.65)	3.63 (2.68–4.92)	3.21 (4)
Tefluthrin	420	2.36 (0.20)	0.85 (0.62–1.19)	3.83 (3.00–4.89)	7.68 (4)
DDT	420	2.98 (0.43)	15.1 (8.81–24.5)	2.00 (1.56–2.57)	14.6 (4)
Methyl Parathion	360	7.54 (1.17)	0.16 (0.13–0.19)	2.63 (2.24–3.09)	3.25 (3)
**Saunders Co-NE (field susceptible)**					
Bifenthrin	420	1.36 (0.17)	1.50 (1.14–2.05)	2.57 (1.84–3.59)	2.79 (4)
Tefluthrin	360	2.85 (0.33)	0.31 (0.15–0.48)	1.40 (1.08–1.80)	7.65 (3)
DDT	540	4.15 (0.67)	9.87 (5.89–12.7)	1.31 (1.02–1.69)	12.1 (6)
Methyl Parathion	360	4.49 (0.60)	0.12 (0.11–0.14)	2.00 (1.67–2.40)	1.08 (3)
**Cache Co-UT (field susceptible)**					
Bifenthrin	360	3.26 (0.32)	0.45 (0.24–0.73)	0.77 (0.61–0.96)	10.4 (3)
Tefluthrin	420	6.73 (1.08)	0.21 (0.18–0.23)	0.93 (0.75–1.15)	0.61 (4)
DDT	360	3.66 (0.47)	4.63 (3.91–5.34)	0.61 (0.48–0.78)	2.12 (3)
Methyl Parathion	360	7.92 (1.23)	0.19 (0.15–0.23)	3.13 (2.67–3.67)	3.41 (3)
**Washington Co-NE (field susceptible)**					
Bifenthrin	360	2.96 (0.39)	0.62 (0.36–1.01)	1.07 (0.83–1.37)	6.11 (3)
Tefluthrin	360	3.19 (0.30)	0.38 (0.27–0.53)	1.71 (1.37–2.16)	5.94 (3)
DDT	360	3.08 (0.39)	4.58 (3.75–5.40)	0.61 (0.47–0.79)	1.35 (3)
Methyl Parathion	360	2.17 (0.22)	0.13 (0.08–0.24)	2.19 (1.76–2.71)	8.48 (3)
**Cuming Co-NE (field ‘moderate’ resistant)**					
Bifenthrin	360	3.47 (0.35)	0.58 (0.30–0.96)	0.99 (0.80–1.23)	12.4 (3)
Tefluthrin	360	3.26 (0.32)	0.83 (0.54–1.45)	3.76 (2.98–4.73)	9.82 (3)
DDT	360	3.50 (0.34)	8.99 (7.85–10.28)	1.19 (0.95–1.50)	2.73 (3)
Methyl Parathion	360	3.94 (0.38)	0.17 (0.11–0.28)	2.82 (2.37–3.36)	10.9 (3)
			**2016**		
**Non-diapause (lab susceptible)**					
Bifenthrin	420	4.02 (0.52)	0.88 (0.79–0.96)	-	0.90 (4)
Tefluthrin	300	8.69 (1.03)	0.45 (0.41–0.48)	-	1.78 (2)
DDT	360	4.75 (0.41)	18.1 (16.4–19.7)	-	2.46 (4)
Methyl Parathion	300	5.48 (0.97)	0.19 (0.17–0.21)	-	1.75 (2)
**Keith Co 2-NE (field resistant)**					
Bifenthrin	420	2.13 (0.19)	1.04 (0.70–1.51)	1.19 (0.97–1.45)	8.49 (4)
Tefluthrin	360	9.10 (0.92)	1.38 (1.17–1.58)	3.11 (2.85–3.39)	9.66 (3)
DDT	420	4.11 (0.42)	26.4 (23.9–28.7)	1.46 (1.28–1.66)	9.33 (4)
Methyl Parathion	360	9.00 (1.32)	0.16 (0.15–0.17)	0.86 (0.76–0.97)	0.76 (3)
**Keith 4 Co-NE (field resistant)**					
Bifenthrin	420	2.31 (0.26)	0.86 (0.35–1.41)	0.98 (0.76–1.25)	12.2 (4)
Tefluthrin	480	6.22 (0.81)	1.63 (1.42–1.83)	3.67 (3.31–4.07)	7.81 (5)
DDT	420	4.66 (0.52)	30.1 (27.0–32.4)	1.66 (1.46–1.90)	2.36 (4)
Methyl Parathion	360	4.07 (0.40)	0.15 (0.13–0.17)	0.78 (0.67–0.91)	0.21 (3)
**Keith 5 Co-NE (field resistant)**					
Bifenthrin	420	3.36 (0.34)	2.17 (1.88–2.49)	2.48 (2.09–2.93)	0.96 (4)
Tefluthrin	420	7.04 (0.86)	1.27 (1.00–1.53)	2.85 (2.57–3.16)	11.8 (4)
DDT	480	6.09 (0.91)	31.4 (24.9–35.4)	1.74 (1.52–1.98)	6.95 (5)
Methyl Parathion	480	5.62 (0.69)	0.22 (0.21–0.24)	1.16 (1.03–1.30)	3.42 (5)

^a^Total number of neonates tested

^b^ LC_50_ RRs calculated between field and non-diapause susceptible populations for each respective year

*Bifenthrin LC_50_ data in Pereira et al. [10]

In 2016, RR_50_s for the three Keith populations collected in 2015 were < 4-fold for the four insecticides when compared to the non-diapause susceptible population (Table 4). In general, the RRs for larvae were considerably lower than adults, especially for tefluthrin (Table 4). This trend was also consistent when larval and adult RR were compared within populations (Table 2).

## Discussion

Results from this present study suggest that multiple mechanisms confer pyrethroid resistance in WCR populations in western Nebraska and Kansas. Diagnostic bioassay data indicate cross-resistance between bifenthrin, tefluthrin and DDT among the field populations tested. Most of the populations in 2014 and 2015 showed reduced susceptibility to these insecticides, especially to tefluthrin. This cross-resistance pattern might suggest that a *kdr*-like mechanism involving insensitivity of the sodium channel is responsible for resistance among those field populations, particularly in southwestern Nebraska and Kansas. This mechanism confers broad cross-resistance among pyrethroids and DDT and has been reported in the Colorado potato beetle, *Leptinotarsa decemlineata* (Say), another Chrysomelid which has also been reported to exhibit cross-resistance among pyrethroids. Studies have documented *kdr* resistance in *L*. *decemlineata* with up to three point mutations in the sodium channel gene in the same population [32, 45].

Synergism bioassays performed with tefluthrin, which showed the highest RRs among the compounds tested (Table 2), also suggest a role for detoxifying enzymes as a resistance mechanism since the RRs in presence of both PBO and DEF were significantly lower. In addition, *kdr* resistance is markedly unaffected by synergists [30]. The synergism results suggest that cytochrome P450s may play a role in the resistance to tefluthrin as evidenced by the significant reduction in RRs when compared to RR in the absence of PBO (Table 3). DEF also reduced RR in the same populations although the reduction was less than PBO, suggesting that hydrolases are also potentially involved in the detoxifying mechanism. Tefluthrin possesses an ester bond and increased hydrolytic metabolism of tefluthrin in WCR has been suggested by Wright et al. [46]. However, it has been suggested that DEF is not a specific hydrolase inhibitor and that it can also inhibit microsomal oxidases at high concentrations [47, 48], which could possibly explain the reduction in RRs in our study. The higher methyl parathion RR recorded from pyrethroid resistant Keith 2 and Keith 4 populations versus the susceptible control populations (Table 2) provides additional support for a possible role for detoxifying enzyme involvement in the resistance mechanism.

Organophosphates and carbamates have been used in Nebraska since early 1960s as replacements for the chlorinated hydrocarbons [18]. A 10-year study in the 1960s and 1970s showed a slight decrease in WCR adult susceptibility to OPs during first five years of survey, and a stabilization of susceptibility afterwards in two of the same Counties (Keith and Chase) where we have tested WCR adult susceptibility [49, 50]. Organophosphate and carbamate resistance in WCR have been previously reported in Nebraska and Kansas [8, 9, 33], and biochemical studies performed to investigate the resistance mechanisms indicated that both P450’s and esterases were involved in OP resistance in WCR [51–55]. Because there has been selection for insecticide resistance with different insecticides in western Nebraska, it may not be unexpected to find multiple mechanisms of resistance in WCR populations.

It is of interest to note that RRs for tefluthrin and DDT were not as high in larval bioassays relative to the corresponding adult populations. RRs for all compounds were < 4-fold and some less than 2-fold. Differences in RRs among insect life stages have been documented in other species [54]. Xi et al. [55] reported different RRs in cotton aphids, *Aphis gossypii* Glover, to the systemic insecticide, spyrotetramat, between adults (579-fold) and nymphs (15-fold) when compared to a susceptible population. Arnold and Whitten [56] observed up to 16-fold higher LC_50’_s in larvae of a resistant strain of *Lucilia cuprina* Wiedemann, to OP when compared to adults. Liu et al. [57] documented higher LC_50_s with 1.5-5-fold differences between susceptible and resistant neonates of diamond back moth, *Plutella xylostella* (L.) exposed to Bt in leaf residues, when compared to third instar larvae, suggesting that physiological tolerance to Bt was higher and feeding behavior was different in neonates when compared to older larvae.

One possible explanation for the general lower RRs in WCR neonates might be related to the use of newly hatched and unfed larvae that may have greatly lowered expression of detoxifying enzymes. Bouvier et al. [58] reported higher ratios and expression of cytochrome P450 monooxygenases and glutathione-S-transferases in late larval instars of resistant Codling moth, *Cydia pomonella* (L.), when compared to early instars. These results might suggest that the higher RRs observed in adult rootworms represent the combined effects of both target site insensitivity and enzymatic detoxification, while the lower RRs observed in neonates are associated with target site insensitivity alone. Future bioassays using fed and unfed first instars may help sort out the observed difference in response to insecticide exposure between adults and larvae.

In this study, WCR adults exhibited RRs < 10-fold for bifenthrin (type I pyrethroid) and RRs as high as 54.8-fold for tefluthrin (type II pyrethroid). Structural and molecular differences between these two pyrethroids may be one of the reasons for the differences in WCR mortalities, especially in adults, when compared to bifenthrin. However, bifenthrin is commonly used as an adulticide in Nebraska [10], whereas tefluthrin is only used as a soil insecticide at planting time for larval control, not only in Nebraska but also in other areas of the U.S. Corn Belt [59–61]. Differences in susceptibility among pesticides of the same classes have been reported previously [62, 63]. Some insecticides from the same class can discriminate completely between susceptible and resistant individuals, whereas dose-response curves for others can overlap making it impossible to separate the resistant individuals [64]. Roush et al. [63] reported differences in susceptibility of horn flies, *Haematobia irritans* (L.), to the pyrethroids cypermethrin (RR: 162-fold) and permethrin (RR: 56-fold). Roush and Daly [54] also found differences in susceptibility to the carbamates carbaryl and propoxur in the predatory mite, *Metaseiulus occidentalis* Muma, and concluded that propoxur produced a much steeper mortality curve and lower slope when compared to carbaryl which produced a shallow mortality curve.

Our results with WCR adults are consistent with other studies investigating cross-resistance in that higher resistance levels were often exhibited in DDT bioassays than in other pyrethroid bioassays. In our case, WCR adult RRs for DDT (up to 33-fold) were higher than recorded with bifenthrin (up to 6.5-fold) but in contrast, only higher than one population (Keith 4) bioassayed with tefluthrin (Table 2). Sawicki [64] reported different RRs when DDT (22-fold) was compared to the pyrethroids bioresmethrin (4.5-fold), cismethrin (14-fold), and decamethrin (18-fold), topically applied to adults of a resistant population of housefly, *Musca domestica* (L.). In contrast to our results, Scott et al. [65] reported differences in susceptibility in resistant German cockroach, *Blattella germanica* (L.), between DDT and type II pyrethroids cypermethrin and deltamethrin when applied using surface-treated methods. They found cross-resistance between DDT and the type I pyrethroids allethrin, permethrin, and fenvalerate. When applied topically to *B*. *germanica*, there was cross-resistance between DDT and all pyrethroids tested [65]. Scott and Matsumura [66] found differences in susceptibility in the *B*. *germanica*, between DDT and type I and type II pyrethroids, showing LC_50_s for DDT 300-fold higher when compared to deltamethrin (type I) in a surface applied bioassay. When topically applied, Scott and Matsumura [66] also found > 1,500-fold higher LD_50_ for DDT when compared to deltamethrin, and > 90-fold higher LD_50_ between allethrin and deltamethrin.

Diagnostic and dose-mortality bioassay data (Fig 1; Table 2) provide further documentation that pyrethroid resistance exists in western Nebraska and Kansas. Adult mortalities at diagnostic concentrations in the susceptible field populations (Saunders County, Scotts Bluff County, and the non-diapause lab population) were > 82% for all insecticides and significantly higher than the other field populations tested (Fig 2). The relative susceptibility of populations to bifenthrin in southwestern areas of Nebraska and Kansas was similar to that reported by Pereira et al. [10]. In southeastern Keith County, Nebraska where a grid of adult populations was collected and bioassayed in 2015, a mosaic of susceptibility to bifenthrin was observed (Fig 1), indicating that significant variation in susceptibility can occur from field to field in a relatively small area. However, because the highest mean proportion bifenthrin mortality was only about 0.60 (Keith 9), all populations included a relatively high frequency of resistant individuals suggesting that resistance is a neighborhood problem in this area. It is unclear if the low level of pyrethroid resistance confirmed in Nebraska and Kansas originated from one or multiple locations. Additional sampling will need to be conducted in the future to document the geographic limits of resistance in Kansas, Nebraska, and neighboring states.

In summary, our results suggest cross-resistance between DDT and tefluthrin in bifenthrin resistant WCR adults. It also appears likely that multiple resistance mechanisms exist, including insensitivity of target site, *kdr*, and metabolic resistance by P450 monooxygenases, with some potential involvement of hydrolytic metabolism. Variable levels of resistance are still present in southwestern areas of Nebraska and Kansas, and it is unclear whether multiple resistance has been selected for a long time with different compounds or if the current pyrethorid resistance has developed in recent years due to increased use of pyrethroids in cornfields when the WCR was either the target or a non-target insect [10]. In addition, differences in susceptibility to pyrethroids between adults and neonate larvae indicate a new finding for WCR, which should stimulate additional research in resistance evolution physiology. Additional research is underway to understand the potential impact of the level of bifenthrin resistance identified in Pereira et al. [10] and this paper when formulated products are applied in the field. Also, molecular studies including sequencing of the sodium channel gene may reveal if there is a point mutation in the sodium channel or if differences in regulation of detoxification enzymes may contribute to resistance.
